# Effect and mechanisms of sacral nerve stimulation on visceral hypersensitivity mediated by nerve growth factor

**DOI:** 10.1111/jcmm.14660

**Published:** 2019-10-22

**Authors:** Liuqin Jiang, Bixing Ye, Yun Wang, Ting Yu, Hairong Xu

**Affiliations:** ^1^ Department of Gastroenterology The First Affiliated Hospital of Nanjing Medical University Nanjing China

**Keywords:** Ca2+, NGF‐mediated visceral sensitivity, protein TRPV1, pTRPV1, SNS

## Abstract

To investigate the efficacy of sacral nerve stimulation (SNS) on nerve growth factor (NGF) mediated visceral sensitivity in normal rat and visceral hypersensitivity model rats. 120 male newborn rats were randomly divided into 6 groups: group A was normal model group; group B ~ F were all sensitized with acetic acid enema and grouped again. Group c2 was given NGF antagonist, d2 group was given NGF agonist, e2 group was given PI3K inhibitor, and f2 group was given PLC‐γ inhibitor. After treatment, the expression of NGF, TrKA, PI3K, AKT, PLC‐γ, NF‐κB, TRPV1, pTRPV1 and intracellular Ca^2+^ content were detected. The expression of protein TRPV1 and pTRPV1 was increased, and Ca^2+^ was increased in the visceral hypersensitive group. NGF, TrKA in NGF antagonist group, PI3K, AKT, NF‐κB in PI3K inhibitor group, PLC‐γ in PLC‐γ inhibitor group were all almost not expressed. The relative expression of NGF, TrKA, PI3K, AKT, PLC‐γ and NF‐κB in NGF antagonist group was lower than that in visceral hypersensitivity group and NGF activator group (*P* < .01). The relative expression of NGF, TrKA, PI3K and AKT mRNA in NGF antagonist group was lower than that in the normal model group (*P* < .01). There was no significant difference in the relative expression of PLC‐γ and NF‐κB mRNA (*P* > .05). The expression level of MAPK, ERK1 and ERK2 in visceral hypersensitivity group was higher than that in PI3K inhibitor group and PLC‐γ inhibitor group. The normal group Ca^2+^ curve was flat, and the NGF agonist group had the highest Ca^2+^ curve peak. Calcium concentration in visceral hypersensitivity group was higher than that in PI3K inhibitor group and that in PLC‐γ inhibitor group was higher than that in NGF antagonist group. The binding of TrkA receptor to NGF activates the MAPK/ERK pathway, the PI3K/Akt pathway and the PLC‐γ pathway, causing changes in the fluidity of intracellular and extracellular Ca^2+^, resulting in increased sensitivity of visceral tissues and organs.

## 1 INTRODUCTION

Irritable bowel syndrome (IBS) is the most common digestive tract disease.^1^ The prevalence of IBS in the general population is 3%‐22%,^2^, ^3^ which seriously affects the quality of life of patients and expends a lot of medical resources. The mechanism of IBS is still unclear. Visceral hypersensitivity is considered to be one of the main cause of IBS. Visceral hypersensitivity is closely related to nerve plasticity in pain pathways of central, peripheral and enteric nervous system (ENS).^4^ At present, the treatment of IBS is mainly to improve the symptoms, the curative effect is not satisfactory, and the symptoms often recur. Exploring the visceral hypersensitivity mechanism of IBS and finding new treatment methods are the research hotspots.

Sacral nerve stimulation (SNS) is a kind of peripheral nerve regulation. It was initially used for the treatment of urinary incontinence and retention. In 1995, it was used by Matzel for the minimally invasive treatment of faecal incontinence.^5^ SNS was more and more widely used in the treatment of bladder dysfunction, faecal incontinence and some intractable constipation because of its minimally invasive, safe, effective and economical characteristics.^6^, ^7^, ^8^ However, the high sensitivity of SNS to IBS or viscera has rarely been reported. Fassov J performed SNS on 21 DIARRHEA‐TYPE IBS patients and found that symptoms of some patients improved after treatment.^9^ Langlois L reported that anorectal dilatation (acute visceral hypersensitivity model) and SNS on normal SD rats could improve their visceral hypersensitivity induced by anorectal dilatation.^10^

Nerve growth factor (NGF) is one member of the neurotrophic factor family. It is widely distributed in the central nervous system, autonomic nervous system and intestinal nervous system. NGF has a stable synaptic nutritional role in regulating the transmission of synaptic signals. NGF mainly binds to two kinds of membrane receptors, usually with high‐affinity receptor tyrosine kinase A (TrkA), regulates downstream signalling pathways, promotes neurotransmitter release, synaptic receptor expression and changes neuroplasticity, and plays an important regulatory role in the survival, growth, differentiation and function of neurons.^11^ It was reported in IBS patients and visceral hypersensitivity animal models; NGF in serum and colon tissue was significantly higher than that in control group. Some scholars found that increased NGF could up‐regulate the expression of TrkA receptor, while NGF antibody could improve visceral sensitivity and down‐regulate the expression of TrkA receptor.^12^, ^13^, ^14^ NGF participates in the visceral hypersensitivity process, which leads to the decrease of visceral pain threshold. It may cause peripheral or central sensitization by regulating neuroplasticity and neurosynaptic transmitter secretion.^4^, ^15^, ^16^ The specific mechanism is still unclear.

In this study, acetic acid enema‐induced chronic visceral hypersensitivity rats were used as animal models. The expression of NGF, TrkA receptor, PI3K/Akt and PLC in colon and DRG before and after SNS were detected by Western blot and RT‐PCR. After antagonizing NGF, the above contents were detected and the effect of SNS on visceral sensitivity was evaluated. The visceral sensitivity of rats was evaluated by blocking the downstream signal pathways separately. To explore whether SNS can improve visceral sensitivity by reducing NGF down‐regulation of TrkA receptor, and further study whether SNS can improve visceral sensitivity by affecting downstream signalling pathways (MAPK/ERK pathway, PI3K/Akt pathway and PLC pathway) through NGF/TrkA receptor. This study will help us to further understand the signal transduction mechanism of visceral hypersensitivity and provide a theoretical basis for the clinical application of neuromodulation in the treatment of IBS.

## 2 MATERIALS AND METHODS

### 2.1 Laboratory animals and groups

One‐Twenty SPF‐grade male newborn rats were fed at quiet environment with room temperature (22 ± 2℃), constant humidity (40%‐50%) and 12/12 hours of circadian circulation. One week after adaptive feeding, 120 healthy and clean SD rats were randomly divided into 6 groups: group A, group B, group C, group D, group E and group F, 20 rats in each group.

Group A: Normal model group.

Group B: The visceral hypersensitivity model was established by acetic acid enema sensitization, and half of the model rats in group B were numbered b1/b2, respectively.

Group C: On the basis of the model group, half of the rats in group C were numbered c1/c2. Group C was given NGF antagonists.

Group D: On the basis of model group, half of the model rats in group D were numbered d1/d2, and group d2 was given NGF agonists.

Group E: On the basis of model group, half of model rats in group E were numbered e1/e2, and PI3K inhibitors were given in group e2.

Group F: On the basis of the model group, half of the rats in group F were numbered f1/f2, and the f2 group was given PLC‐γ inhibitor.

### 2.2 Method

At the age of 10 days, the neonatal rats were fed with 0.5% acetic acid solution 0.2 mL through intestinal perfusion with insertion of anus for 2 cm, while the neonatal rats in group A were fed with 0.2 mL saline through intestinal perfusion. Electrode implantation was performed at the age of 7 weeks in rats, and the experiment began at the age of 8‐12 weeks.

The expression of TRPV1 and pTRPV1 in dorsal root ganglion and colon tissues of group A and group b1 were detected by Western blot (WB). The content of Ca^2+^ in the cells of dorsal calcaneal ganglion and colon was detected by fluorescence spectrophotometer with fura‐2 indicator. The expressions of NGF, TrKA, PI3K, AKT, PLC‐γ, NF‐κ B, TRPV1 and pTRPV1 in dorsal root ganglion and colon tissues of animals in groups A, b2, c2, d2, E2 and F2 were detected by WB. Expressions of NGF, TrKA, PI3K, AKT, PLC‐γ, NF‐κ B, TRPV1 and pTRPV1 were analysed by real‐time fluorescence quantitative polymerase chain reaction (RT‐PCR). Co‐Immunoprecipitation (CO‐IP) was used to analyse the interaction between NGF and TrK A in dorsal root ganglion and colon tissues of animals in groups A, b2, C2 and d2, and fluorescence spectrophotometer was used to detect the intracellular Ca^2+^ content in dorsal heel ganglion and colon tissues of animals in groups A, b2, c2, d2, E2 and f2.

### 2.3 Statistical methods

SPSS 18.0 statistical software was used to process and analyse the data, and the measurement data were described by mean ± standard deviation. Single factor analysis of variance was used for multi‐group comparison, and correlation analysis was used for correlation analysis. *P* < .05 was statistically significant.

## 3 RESULT

### 3.1 Expression of TRPV1 and pTRPV 1 in rats of group A and group b1

**Figure 1:**
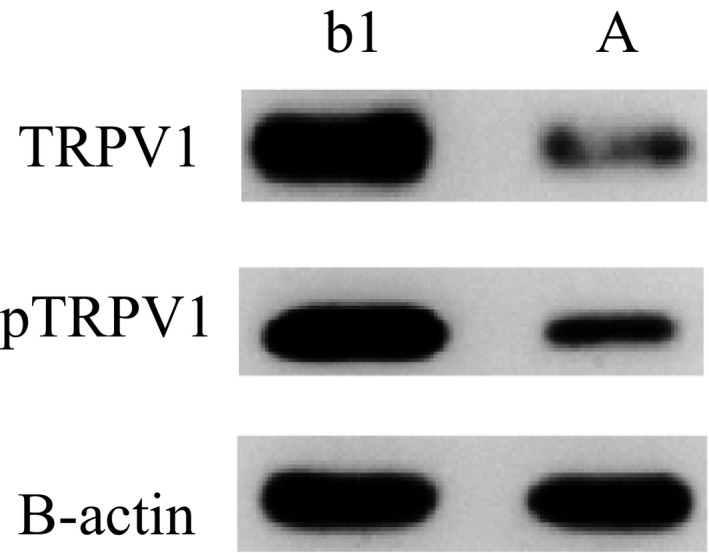
Expression of TRPV1 and pTRPV1 in rats of group A and group b1

As shown in Figure 1, the expression of TRPV1 and pTRPV1 was high in group b1 (visceral hypersensitivity group), and the expression of TRPV1 and pTRPV1 was low in group A (normal non‐model group).

### 3.2 Intracellular Ca^2+^ content in dorsal calcaneal ganglion and colon tissues of rats in group 2A and group b1

**Figure 2:**
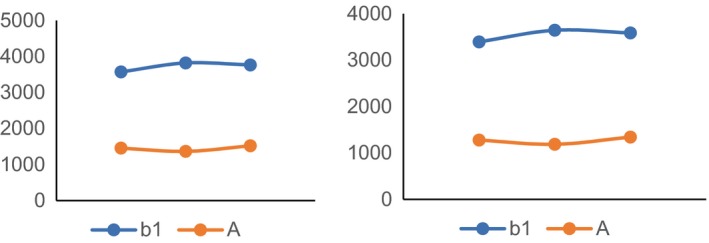
The intracellular Ca^2+^ content in dorsal calcaneal ganglion and colon tissue

The content of Ca^2+^ in dorsal ganglion and colon tissue cells was detected by fluorescence spectrophotometer with fura‐2 indicator. The left figure in Figure 2 shows the intracellular Ca^2+^ content in dorsal calcaneal ganglion cells, and the right figure shows the intracellular Ca^2+^ content in colonic tissue cells. In both dorsal ganglion and colon tissues, the intracellular Ca^2+^ content in group b1 (visceral hypersensitivity group) was higher than that in group A (normal non‐model group).

### 3.3 Expression of NGF, TrKA, PI3K, AKT, PLC‐γ, NF‐κB, TRPV1 and pTRPV1 in rats of group A, group b2, group c2, group d2, group e2 and group F2

**Figure 3:**
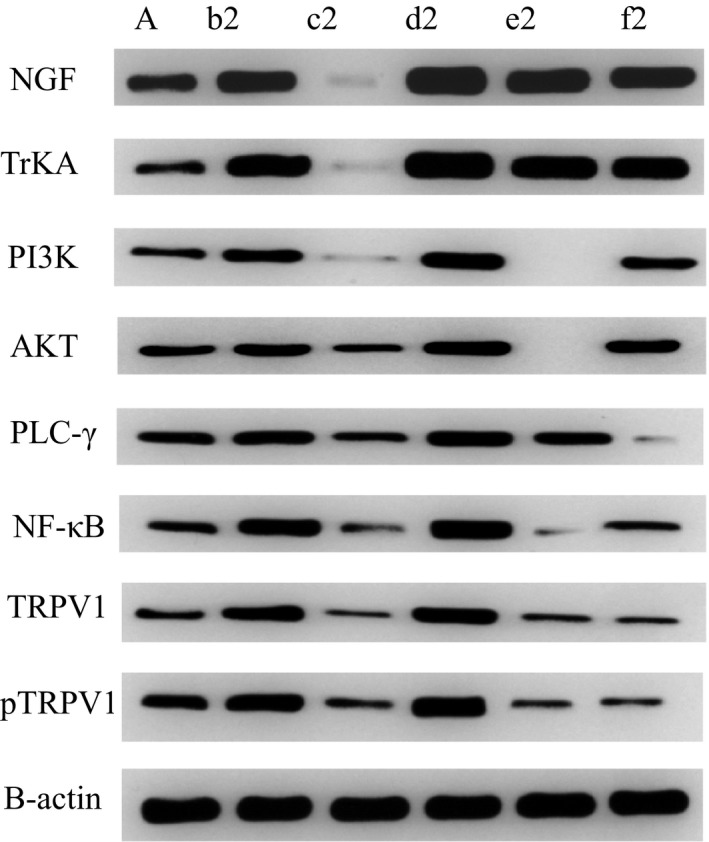
Protein expression in rats of each group

As shown in Figure 3, the expression of NGF, TrKA, PI3K, AKT, PLC‐γ, NF‐κB, TRPV1 and pTRPV1 in group A (normal non‐model group) was minimal. NGF, TrKA, PI3K, AKT, PLC‐γ, NF‐κB, TRPV1 and pTRPV1 were highly expressed in group b2 (visceral hypersensitivity group). In group c2 (NGF antagonist group), protein NGF and TrKA were almost not expressed, while other proteins were microexpressed. The expression of NGF, TrKA, PI3K, AKT, PLC‐γ, NF‐κB, TRPV1 and pTRPV1 in group d2 (NGF agonist group) was higher than that in other groups. In the e2 group (PI3K inhibitor group), PI3K, AKT and NF‐κB were almost not expressed, while other proteins were microexpressed. In f2 group (PLC‐γ inhibitor group), protein PLC‐γ was almost not expressed, while other proteins were microexpressed.

### 3.4 Gene expression of NGF, TrKA, PI3K, AKT, PLC‐γ and NF‐κB in rats of group 4A, group b2, group c2 and group d2

**Figure 4:**
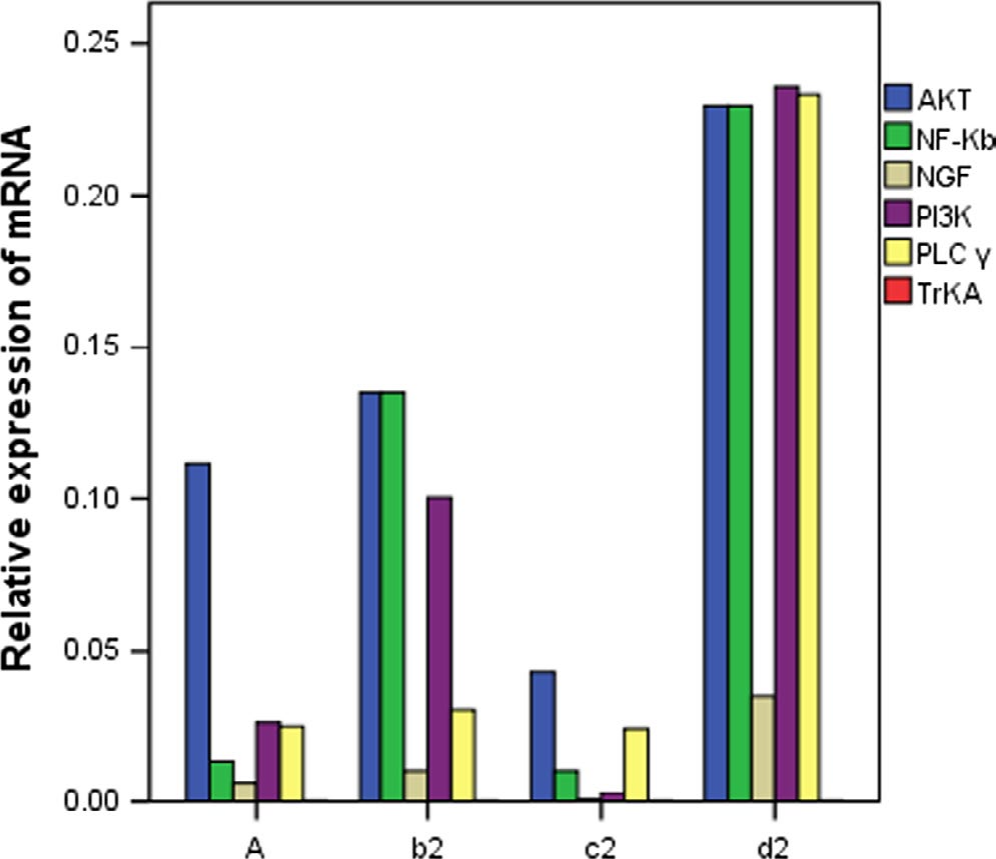
Relative expression of mRNA in each group of rats

RT‐PCR was used to analyse the expression of the target gene. The relative expression of the target gene was analysed by CT. Normal or negative samples were usually used as control samples. The relative expression of NGF, TrKA, PI3K, AKT, PLC‐γ and NF‐κB was 2^−ΔΔCT^, ΔCT was the target gene CT value‐internal reference (GAPDH) CT value, and ΔΔCT was the target sample ΔCT‐control sample ΔCT. The expression levels of NGF, TrKA, PI3K, AKT, PLC‐γ and NF‐κB in different groups were analysed by one‐way ANOVA. The results in Figure 4 and Table 1 showed that the relative expression levels of NGF, TrKA, PI3K, AKT, PLC‐γ and NF‐κ B in each group were significantly different (*P* < .01). The results of LSD showed that there was no significant difference in the relative expression of PLC‐γ and NF‐κ B between group A (normal non‐model group) and group c2 (NGF antagonist group; *P* > .05). There was no significant difference in the relative expression of PLC‐γ between group A (normal non‐model group) and group b2 (visceral hypersensitivity group; *P* = .08 > .05). There was no significant difference in the relative expression of PLC‐γ between the other two groups (*P* = .08 > .05). The difference was statistically significant (*P* < .05).

**Table 1:** Relative expression of mRNA in rats of each group (X ± S)

Group	NGF	TrKA	PI3K	AKT	PLC‐γ	NF‐κB
A	0.007 ± 0.000	0.0002 ± 0.000	0.027 ± 0.002	0.111 ± 0.010	0.025 ± 0.002	0.014 ± 0.001
b2	0.011 ± 0.001	0.0001 ± 0.001	0.100 ± 0.30	0.13 ± 0.008	0.031 ± 0.002	0.135 ± 0.008
c2	0.001 ± 0.000	0.0002 ± 0.000	0.003 ± 0.000	0.432 ± 0.022	0.025 ± 0.001	0.108 ± 0.001
d2	0.035 ± 0.006	0.0002 ± 0.000	0.236 ± 0.014	0.230 ± 0.009	0.233 ± 0.012	0.230 ± 0.009
F	206.880	90.567	351.608	863.962	2428.062	2708.031
P	0.000	0.000	0.000	0.000	0.000	0.000

### 3.5 Expressions of MAPK, ERK1 and ERK2 in rats of 5b2, e2 and f2 groups

**Figure 5:**
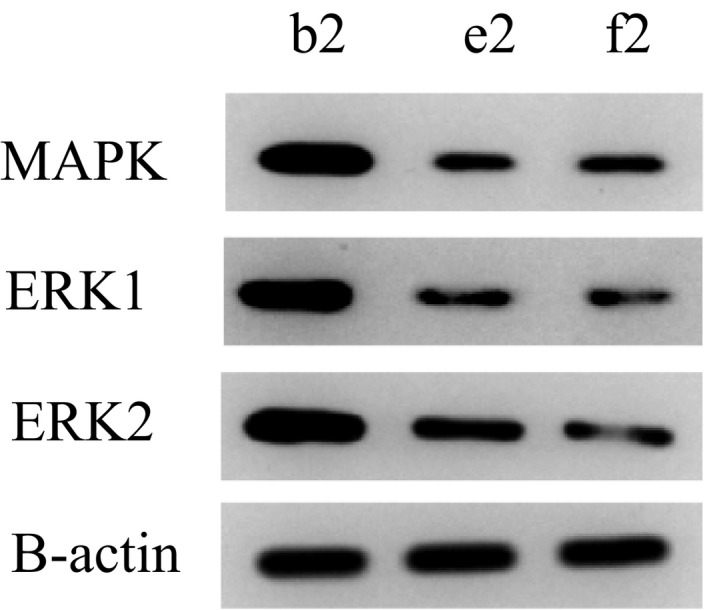
Expression of MAPK, ERK1 and ERK2 in rats

As shown in Figure 5, the expression levels of MAPK, ERK1 and ERK2 in group b2 (visceral hypersensitivity group) were higher than those in group e2 (PI3K inhibitor group) and group f2 (PLC‐γ inhibitor group). The expression levels of MAPK, ERK1 and ERK2 in group e2 were similar to that in group f2.

### 3.6 Intracellular Ca^2+^ content in rats of group 6A, group b2, group c2, group d2, group e2 and group f2

**Figure 6:**
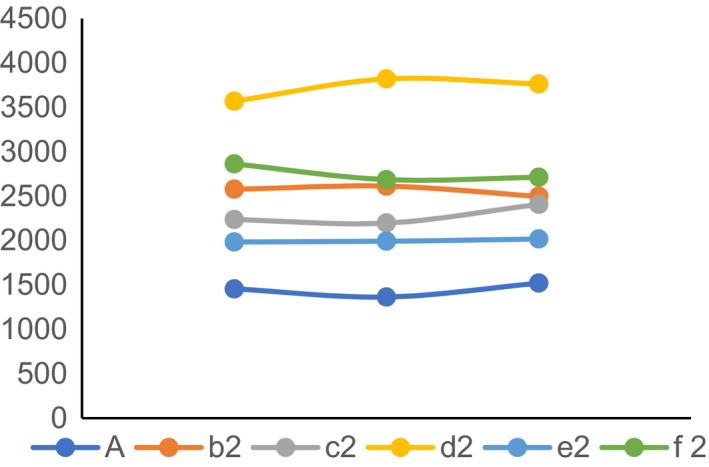
Ca^2+^ content in rat dorsal calcaneal ganglion cells

**Figure 7:**
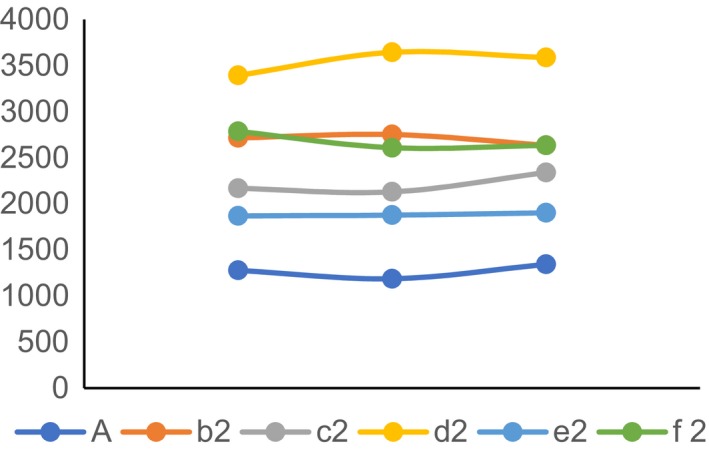
The intracellular Ca^2+^ content in rat colon tissue

The content of Ca^2+^ in dorsal calcaneal ganglion and colon tissue cells was measured by fluorescence spectrophotometer with fura‐2 indicator. As shown in Figures 6 and 7, the curve of Ca^2+^ in both dorsal calcaneal ganglion and colon tissue cells was flat in the normal group in Figure 6. The curve peaks in group d2 (NGF agonist group) were the highest, while those in other groups were b2 (visceral hypersensitivity group) higher than that in e2 (PI3K inhibitor group) and f2 (visceral hypersensitivity group). Group A (PLC‐γ inhibitor group) higher than that in group c2 (NGF antagonist group).

## 4 DISCUSSION

Visceral hypersensitivity (VH) refers to the increased responsiveness and sensitivity of visceral tissues to various endogenous or exogenous chemical or physical stimuli. Increased visceral sensitivity is one of the main pathophysiological mechanisms of IBS‐D and is an important cause of abdominal pain, gastrointestinal motility disorders and clinical symptoms. Nonnoxious and harmful receptors are widely distributed in various organs of the body. The intestinal wall receptor can be directly contacted with the contents of the intestinal cavity. When the intestinal wall receptor is stimulated by noxious substances (intestinal contents), the intestinal wall receptor transmits the stimulus signal along the afferent nerve to the dorsal root ganglion, which is regulated by the central visceral sensory region to produce visceral pain.

NGF is a kind of protein that can induce the growth of menstruation and is closely related to the development of nervous system.^17^ In recent years, it has been found that nerve growth factor also plays an important role in the generation and maintenance of body pain.^18^ As one of the neurotrophic factors, NGF can be synthesized and released by a variety of cells, including nerve cells, tissue cells and inflammatory cells.^19^ NGF binds to protein tyrosine kinase receptor A (TrKA) and tumour necrosis factor (TNF) receptor p75NTR, and tyrosine kinase receptor A (TrKA) is a high‐affinity receptor of NGF.^20^ TrKA is expressed in peripheral nervous system, central nervous system and various organs and tissues of the whole body, such as digestive tract, kidney, skin and immune tissue. TrkA receptor binds to NGF to activate intracellular signalling cascades, further regulate nerve junction function, inhibit or protect nerve cells, promote neurotransmitter release, neuroreceptor expression and alter neuroplasticity, including 21, 22: (1) MAPK/ERK pathway, activation of small molecular guanosine‐binding protein‐guanosine diphosphate (Ras‐GDP) into Ras‐guanosine triphosphate (Ras‐GDP). S‐GTP, which causes the phosphorylation of MAPK kinase (MEK), activates MAPK and activates the expression of cell survival promoting factor (Bcl‐2); (2) PI3K/Akt pathway, phosphatidylinositol‐3‐kinase (PI3K) is activated after it is connected with phosphorylated TrkA residues, activates IAP and Akt, further activates CREB and increases the transcription of Bcl‐2; (3) phospholipase C (PLC) ‐γ pathway is activated, and PLC is produced after activation. Diacylglycerol (DAG) and inositol triphosphate (IP3), which cause the release of internal calcium (Ca^2+^), further activate protein kinase C (PKC) and play its catalytic role in protein phosphorylation.^23^, ^24^

Previous studies have reported that PI3K/Akt and PLC signalling pathways play an important role in TNBS‐induced visceral hypersensitivity in rats, possibly through up‐regulating neuroreceptors such as NMDA receptors, promoting the release of SP, CGRP and other peptide transmitters, and altering neuroplasticity involved in the process of spinal sensitization to visceral hypersensitivity. The results of this study also confirm this point.

In this study, we found that the expression of TRPV1 and pTRPV1 and the concentration of Ca^2+^ increased in visceral hypersensitivity group. NGF antagonist was given to visceral hypersensitive rats, and protein NGF and TrKA were hardly expressed. PI3K inhibitor was given, and protein PI3K, AKT and NF‐κ B were hardly expressed. The relative expressions of NGF, TrKA, PI3K, AKT, PLC‐γ and NF‐κ B in NGF antagonist group were lower than those in visceral hypersensitivity group and NGF activator group (*P* < .01). The expression levels of MAPK, ERK1 and ERK2 in visceral hypersensitivity group were higher than those in PI3K inhibitor group and PLC‐γ inhibitor group. The Ca^2+^ curve of normal group was flat, and the peak of Ca^2+^ curve of NGF agonist group was the highest. It has also been reported that PI3K/Akt and PLC signalling pathways play an important role in TNBS‐induced visceral hypersensitivity in rats. They may participate in visceral hypersensitivity by up‐regulating neuroreceptors such as NMDA receptors, promoting the release of SP and CGRP, and altering neuroplasticity. It is also found that MAPK/ERK signalling pathway does not participate in spinal cord hypersensitivity by up‐regulating the expression of NMDA receptors.

To sum up, the visceral hypersensitivity model was established by acetic acid enema sensitization. Intestinal tissues and intestinal nervous system were sensitized by external noxious stimuli. Intestinal nerve cells, intestinal epithelial cells and intestinal smooth muscle cells were activated to produce and release NGF. NGF combined with TrKA mainly caused to the opening of transport channel TRPV1 through PI3K/Akt and PLC signalling pathways, and the fluidity of intracellular and extracellular Ca^2+^ was changed. In addition, tissue damage can lead to electrolyte disturbance inside and outside the cell, which can also lead to the opening of transport channel TRPV1, the change of fluidity of intracellular and extracellular Ca^2+^ and the increase of the sensitivity of visceral tissues and organs.

## CONFLICT OF INTEREST

All of the authors have no conflict of interest in this research.

## AUTHOR’S CONTRIBUTION

Each author has made an important scientific contribution to the study and has assisted with the drafting or revising of the manuscript.

## SUMMARY

TrkA receptors bind to NGF by activating the MAPK/ERK pathway, PI3K/Akt pathway and PLC‐γ pathway, causing changes in intracellular and extracellular Ca^2+^ fluidity and increasing visceral organ sensitivity.

## ETHICS, CONSENT AND PERMISSIONS

Ethical approval was given by The First Affiliated Hospital of Nanjing Medical University.

## CONSENT TO PUBLISH

All of the authors have consented to publish this research.

## Data Availability

The data are free access to available upon request.
